# Colchicine Ameliorates 5-Fluorouracil-Induced Cardiotoxicity in Rats

**DOI:** 10.1155/2022/6194532

**Published:** 2022-01-28

**Authors:** Soheila Safarpour, Samaneh Safarpour, Marzieh Pirzadeh, Ali Akbar Moghadamnia, Anahita Ebrahimpour, Fatemeh Shirafkan, Razieh Mansoori, Sohrab Kazemi, Mohammad Hosseini

**Affiliations:** ^1^Student Research Committee, Babol University of Medical Sciences, Babol, Iran; ^2^Department of Pharmacology and Toxicology, School of Medicine, Babol University of Medical Sciences, Babol, Iran; ^3^Department of Biochemistry, School of Medicine, Mashhad University of Medical Sciences, Mashhad, Iran; ^4^Cellular and Molecular Biology Research Center, Health Research Institute, Babol University of Medical Sciences, Babol, Iran; ^5^Department of Veterinary Pathology, Babol-Branch, Islamic Azad University, Babol, Iran

## Abstract

*Background and Objective*. 5-Fluorouracil is one of the most common chemotherapeutic agents used in the treatment of solid tumors. 5-Fluorouracil-associated cardiotoxicity is the second cause of cardiotoxicity induced by chemotherapeutic drugs after anthracyclines. Colchicine is a strong anti-inflammatory drug used to prevent and treat acute gout and treat familial Mediterranean fever. And also, its protective effects on cardiovascular disease have been reported in various studies. The current study is aimed at appraising the effect of colchicine on 5-fluorouracil-induced cardiotoxicity in rats. *Methods*. Twenty male Wistar rats were divided into four groups as follows: control, 5-fluorouracil, colchicine (5 mg/kg), and 5-fluorouracil+5 mg/kg colchicine. Cardiotoxicity was induced with an intraperitoneal injection of a single dose of 5-fluorouracil (100 mg/kg). The control group received normal saline, and the treatment groups received colchicine with an intraperitoneal injection for 14 days. *Findings*. 5-Fluorouracil resulted in significant cardiotoxicity represented by an increase in cardiac enzymes, malondialdehyde levels, cyclooxygenase-2 and tumor necrosis factor-alpha expression, cardiac enzymes, and histopathological degenerations. 5-Fluorouracil treatment also decreased body weight, total antioxidant capacity and catalase values, blood cells, and hemoglobin levels. In addition, 5-fluorouracil disrupted electrocardiographic parameters, including increased elevation in the ST segment and increased QRS duration. Treatment with colchicine reduced oxidative stress, cardiac enzymes, histopathological degenerations, and cyclooxygenase-2 expression in cardiac tissue, improved electrocardiographic disorders, and enhanced the number of blood cells and total antioxidant capacity levels. Moreover, body weight loss was hampered after treatment with colchicine. Our results demonstrated that treatment with colchicine significantly improved cardiotoxicity induced by 5-fluorouracil in rats.

## 1. Introduction

Pyrimidine 5-fluorouracil (5-FU) is one of the most broadly used chemotherapeutic drugs through intravenous injection to treat a variety of cancers, especially cancer of the gastrointestinal tract, skin, and breast [1, 2]. 5-FU exerts its anticancer impacts in a number of ways, including inhibition of the thymidylate synthase enzyme [3, 4] and activation of protein p53, as well as an effect on cell cycle regulation and G1/S arrest [5].

Apart from its benefits, this drug leads to implications in a range of patients' organs undergoing chemotherapy, such as myelosuppression, emesis, mucositis, nausea, and toxicity to other organs, mainly the heart [6]. Although 5-FU-induced cardiotoxicity is rare and infrequent, however, it can potentially cause arrhythmias, myocardial ischemia, or heart failure, which presents with symptoms such as chest pain, hypotension, and dyspnoea [7, 8]. Several plausible mechanisms for cardiotoxicity enhanced via 5-FU like thrombogenic effects, coronary artery spasm, and endothelial damage are considered. However, the exact mechanism of cardiac toxicity induced via 5-fluorouracil has not been delineated [9].

Colchicine (COL) is an extract from the Colchicum autumnale plant (autumn crocus) found in corn, seeds, and flowers. Its application as a medicinal plant to treat joint pain was well-documented [10]. Actually, COL is a wide-ranging disposable, low-cost, and heretofore strong anti-inflammatory drug used to prevent and treat acute gout and treat familial Mediterranean fever (FMF) and other inflammatory conditions, such as postpericardiotomy syndrome and pericarditis as well [11, 12]. The mechanism of action in COL is not fully understood. Early studies identified the microtubule as the primary subcellular target; for this reason, COL is generally used at in vitro experimentations requiring cytoskeleton disruption [13, 14].

It can hinder the neutrophil infiltration in the heart of rats after acute myocardial infarction [15, 16]. It suppresses secreting IL-1*β* in patients with acute coronary syndrome as well [17].

Therefore, cardiovascular diseases have an inflammatory component and inflammation is an important component in the progression of heart attacks, and the belief that inflammation causes heart disease has been around for many years. There are many studies on the effect of COL as a strong anti-inflammatory on inflammatory diseases, and some recent studies have shown that COL may have positive effects on heart disease [15, 16, 18].

Hence, our study is aimed at investigating the protective role of COL in 5-FU-induced cardiotoxicity in male rats by appraising the electrocardiographic (ECG), pathological, biochemical, and molecular markers such as cyclooxygenase-2 (COX-2) and tumor necrosis factor-alpha (TNF-*α*) in rat heart tissues.

## 2. Materials and Methods

### 2.1. Chemicals and Reagents

We obtained 5-FU from Sigma, St. Louis, MO (USA). COL was purchased from Merck Company, Germany. Malondialdehyde (MDA), total antioxidant capacity (TAC), and catalase (CAT) kits were prepared from Teb Pajohan Razi Company, Iran. RNA extraction and cDNA synthesis kits were purchased from Sinacolon and Yekta Tajhiz Azma companies, Tehran, Iran, respectively.

### 2.2. Animals

In this study, twenty male Wistar rats in the weight range of 180 ± 20 g and with an approximate age of 6–8 weeks were selected from the animal house of Babol University of Medical Sciences (Babol, Iran). The animals were embedded in cages under controlled temperature (23 ± 2°C) and humidity (60% ± 5%), provided tap water ad libitum, and fed a laboratory animal standard pelletized diet according to the guidelines of the Research Council of Babol University of Medical Sciences. The lighting cycle was 12 hours' light/dark. Then, they were divided randomly into 4 groups of 5 rats each group, and afterward, they were kept in individual wire-bottomed cages.

### 2.3. Treatment

The animals were injected intraperitoneally (IP) [19] for two weeks [20] and accidentally divided into 4 groups as follows:

1. Control group (*n* = 5): animals received normal saline (phosphate-buffered saline) for 14 days
2. COL group (*n* = 5): animals received COL at a dose of 5 mg/kg body weight as IP for 14 days [21]
3. The 5-FU group (*n* = 5): animals received IP injection of a single dose of 5-FU (100 mg/kg) only on the first day of treatment [22, 23]
4. The group received 5-FU at a single dose of 100 mg/kg body weight only on the first day of treatment, and no 5-FU injection was performed from the second day. From day 2 onwards alone, rats were injected with COL at a dose of 5 mg/kg body weight as IP for 14 days (*n* = 5)

The rats in all groups were weighed before and after injection and at the end of the treatment period (two weeks after the first injection). The ECG was recorded. Also, the weight of rats' hearts was measured in all groups. Eventually, they were killed with an overdose of ether, and about 2 mL of blood was taken from the heart, and the serum and heart tissue were separated for evaluation of biochemical, molecular, and histopathological analysis.

### 2.4. Evaluation of ECG Alterations

The ECG was recorded twenty-four hours after the last treatment. All animals were anesthetized with ketamine and xylazine (75/25 mg/kg, i.p.), subcutaneous peripheral limb electrodes were inserted for standard limb lead II recording, and ECG parameters such as ST-elevation, QTc duration, QRS duration, RR interval, and heart rate (HR) with the use of electrocardiographs (ewave 8b, Sciencebeam, Tehran, Iran) were measured.

### 2.5. The Mensuration of Body Weight and Relative Heart Weight

The rats were weighed on the first day, before injection. Weight was also taken on day 14 after injection to compare weight changes between the first day and the 14^th^ day. In addition, the relative weight of rats' hearts was measured by a digital scale as follows:

(1) $$\begin{array}{c} \text{Relative heart weight}=\frac{\text{the heart weight}}{\text{the rat weight}}\times 100\%. \end{array}$$

### 2.6. The Preparation of Serum and Heart Samples

Eventually, after performing various treatments, ether inhalation solution was used to kill. After weighing, heart tissue was removed through the incision for hematoxylin-eosin (H&E) staining, molecular tests, and measured biochemical agents: MDA, CAT, and TAC.

### 2.7. Blood Collection and Evaluation

Blood samples were collected. Blood cells including red blood cells (RBC), white blood cells (WBC), and platelets (PLT) were counted, and the hemoglobin (Hgb) level was measured. Also, the levels of cardiac enzymes including lactate dehydrogenase (LDH), creatine kinase myocardial band (CK-MB), and aspartate transaminase (AST) were evaluated.

The heart tissues were removed from the body and divided into several parts. In each group of hearts, a portion was immediately transferred to the microtube and stored at -80°C until biochemical parameters were evaluated. The part was isolated for tissue cutting, and part of the tissue was considered for molecular tests.

### 2.8. Thiobarbituric Acid Reactive Substance (TBARS) Assay

Briefly, after anesthetizing animals and collecting blood, the part of the heart tissue in each group was isolated and immediately transferred to the microtube and was stored at -80°C until biochemical parameters were evaluated. The TBARS assay has been employed to assess oxidative stress levels in the heart tissue. To measure TBARS levels, 1 mL of tissue was added to 2 mL of TBARS reagent and heated at 100°C for 60 minutes. Then, samples were put in a 10-minute ice bath and then centrifuged for 10 minutes at 2500 rpm. At this phase, thiobarbituric acid will react with MDA to create a red product. The light absorbance was measured via an Elisa reader at 535 nm [24].

### 2.9. Ferric Reducing Antioxidant Power (FRAP) Assay

The antioxidant capacity of the samples was assessed by the FRAP method. After anesthetizing the animals, the heart tissue was removed. Each part of the heart tissue was homogenized to the same weight ratio in 0.5 *μ*L normal saline and after centrifuging for 5 min at 1000 g; the resulting supernatant was analyzed for biochemical assays. Then, we added 1.5 mL of the ready-to-use FRAP reagent to all the tubes and incubated them at 5°C for 37 minutes. Afterward, 51 *μ*L of the sample (tissue extracts or different standards) was added to the tubes and mixed well, and the mixtures were reincubated at 37°C for 15 minutes. This method evaluated the level of antioxidants in each sample through reducing ferric ion (Fe^3+^) to ferro (Fe^2+^). Following ferric iron reduction, the blue color intensity was measured using an Elisa reader at 593 nm [25].

### 2.10. Catalase (CAT) Assay

After anesthetizing rats, the part of the heart tissue was isolated and was stored at -80°C for biochemical parameters were evaluated. The CAT activity was evaluated as previously mentioned by Aebi [26] using the Teb Pajohan Razi Kit, Iran. This process is according to a decrease in absorbance at 240 nm owing to H_2_O_2_ dismutation. To determine the CAT activity, the molar extinction coefficient of H_2_O_2_, 43.6 M cm^−1^, was used. Under standard circumstances, the number of H_2_O_2_ converted into H_2_O and 1/2 O_2_ in 1 min is defined as one unit.

### 2.11. Histopathological Assessment

Part of the heart tissue in each group was instantly collected and inflated with mild PBS. After the fixing process in 4% paraformaldehyde for 24 hours, samples were placed in paraffin, and sections (5 *μ*m) were prepared using a microtome device (model Leitz 1512, Germany). For each slide, an average of three fields was considered.

#### 2.11.1. Hematoxylin and Eosin (H&E) Staining

H&E staining was employed to measure the histopathological degeneration in the heart tissue. For each group, three animals and from each animal, seven slides and in each slide, three fields were selected, and the amount of inflammation was estimated. For histological studies, data analysis was performed by an expert pathologist using ImageJ software.

Briefly, the coloring steps are as follows: placing tissue incisions in 100% alcohol (five minutes); placing tissue incisions in 96% alcohol (five minutes); staining with hematoxylin (five minutes); rinsing with running water (five minutes); eosin staining (15 seconds); immersing in distilled water for decolorization; placing in ethanol 70% (15 seconds), ethanol 95% (30 seconds), absolute ethanol (one minute), and xylene (five minutes); and pasting with Entellan.

H&E staining was used to appraise histology, morphological deformation, and cell inflammation in heart tissue. Cardiac tissue was evaluated under a microscope (Olympus BX61VS, Japan). Then, data analysis was conducted by a pathologist using ImageJ software.

### 2.12. RNA Extraction and Real-Time Polymerase Chain Reaction (RT-PCR)

To evaluate TNF-*α* and COX-2 genes, part of the heart tissue was considered for molecular tests. The heart samples were immediately transferred to microtubes of DNase & RNase free, and the samples were stored in a -20°C freezer overnight; then, the liquid was removed and tissues were transferred to a -80°C freezer and stored until RNA extraction. Then, the total RNA was extracted based on the total RNA extraction kit protocol (Pars Tous, Mashhad, Iran). Also, cDNA was synthesized according to the manufacturer's protocol (Pars Tous, Mashhad, Iran). qRT-PCR was conducted using an ABI Step One Plus Real-Time PCR System (Applied Biosystem, USA) with primer sets for COX-2 and TNF-*α* as target genes and GAPDH as a housekeeping gene. 10 *μ*L real-time PCR reaction mixture comprised of 1 *μ*L cDNA, 6.25 *μ*L SYBR-Green (Amplicon high Rox master mix, Denmark), 2.25 *μ*L nuclease-free water, and 0.25 *μ*L of 10 pmol of each primer (Robin Teb Gostar, Tehran, Iran).

According to Kirkpatrick et al. [27], conditions for the reverse transcription step were 25°C for 10 min, 37°C for 60 min, and 85°C for 5 min. The polymerase chain reaction was carried out by holding temperature for 15 min at 95°C and after that 40 cycles of 15 s at 95°C, 30 s at 62°C, and 30 s at 72°C followed by melting curve temperature steps. The COX-2 and TNF-*α* primers are represented in Table 1.

### 2.13. Statistical Analysis

In this study, the ECG signals were recorded and evaluated using eProbe software. Then, all data were analyzed using GraphPad Prism software version 8. The parametric variables were displayed as the mean ± SD and the nonparametric ones as the median (min–max). We performed a one-way analysis of variance (ANOVA) followed by the Tukey posttest to analyze the results of the ECG parameters, blood count, biochemical assays, and molecular data. In addition, Kruskal-Wallis and Mann–Whitney *U* tests were utilized for histopathological scoring differences between the groups. *P* values less than 0.05 were considered statistically significant.

## 3. Result

### 3.1. Effect of COL on Electrocardiographic Parameters

**Figure 1:**
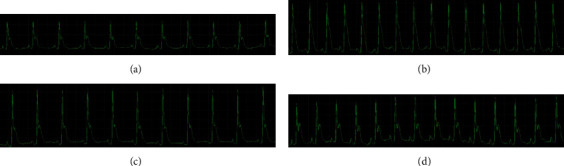
ECG recording from (a) control, (b) 5-FU, (c) COL, and (d) COL+5-FU. (a, c) ECG alterations include ST segment, QTc duration, QRS duration, RR interval, and HR: normal. (b) ECG alterations include ST segment, QTc duration, QRS duration, RR interval, and HR: abnormal compared to the control and COL groups. (d) ECG alterations include a decreased ST segment and decreased QRS duration COL+5-FU group compared to the 5-FU group (number of animals in each group: 5).

The ECG recording (Figure 1) depicted the 5-FU-induced abnormalities in cardiac electrophysiology (Figure 1(b)). It caused elevation in the ST segment compared to the control (*P* < 0.001) (Figure 1(a)) and COL (*P* < 0.01) (Figure 1(c)) groups. It also significantly raised the duration of QRS compared to the control and COL (*P* < 0.001, *P* < 0.001) groups, while having no significant effect on QTc, HR duration, and RR interval compared to the ECG of normal control rats (Table 2).

In the case of the COL+5-FU group, a slight increase in the ST segment was found compared to the control group (*P* < 0.01) (Figure 1(d)). However, a significant decrease in the ST segment was also observed compared to the 5-FU group (*P* < 0.05). On the other hand, the COL+5-FU group also showed a considerable decrease in QRS duration compared to the control, COL, and 5-FU groups (*P* < 0.001, *P* < 0.01, and *P* < 0.001, respectively) (Table 2), while having no significant effect on QTc and HR duration and RR interval compared to the ECG of other treatment groups.

### 3.2. Body Weight Variation and Relative Heart Weight

Body weight was measured on the first day of injection and the last day of injection, the 14^th^ day. Changes in body weight were evaluated and compared between the treated and control groups. The results showed that 5-FU inhibited weight gain compared with the control (*P* < 0.001) and COL groups (*P* < 0.001) significantly, which indicates growth retardation. In addition, a significant reduction in body weight was found in the group receiving COL+5-FU (*P* < 0.001, *P* < 0.001) compared to the control and COL groups.

**Figure 2:**
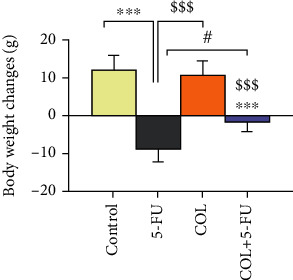
Comparison of the body weight changes in rats of different groups on the first day before injection and the 14^th^ day after injection. All results are expressed as the mean ± SD. ^∗∗∗^*P* < 0.001: significant compared to the control group. ^$$$^*P* < 0.001: significant compared to the COL group. ^#^*P* < 0.05: significant compared to the 5-FU group. SD =standard deviation (number of animals in each group: 5).

However, COL was able to prevent the weight loss of rats treated with 5-FU to some extent, and the weight difference with the 5-FU group was statistically significant in rats receiving COL+5-FU (*P* < 0.05) (Figure 2).

Furthermore, despite the difference in the relative heart weight in the 5-FU group compared to the control (*P* < 0.01) and COL (*P* < 0.05) groups, no significant change in the relative heart weight was detected in the COL+5-FU group (Table 3).

### 3.3. The Effect of COL on Hematological Parameters and Cardiac Enzymes

**Figure 3:**
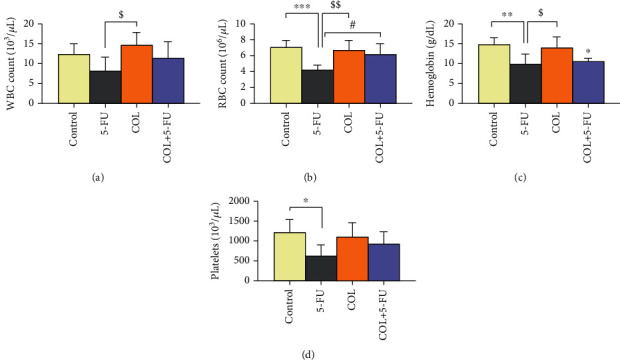
Data quantification indicates the effect of treated groups on blood factors and the comparison of different groups with each other. ^∗^*P* < 0.05,  ^∗∗^*P* < 0.01, and^∗∗∗^*P* < 0.001: significant compared to the control group. ^$^*P* < 0.05, ^$$^*P* < 0.01: significant compared to the COL group. ^#^*P* < 0.05: significant compared to the 5-FU group. All results are expressed as the mean ± SD. WBC = white blood cell; RBC = red blood cell; SD = standard deviation (number of animals in each group: 5).

5-FU administration significantly reduced WBC compared to the COL group (*P* < 0.05). Also, 5-FU significantly reduced RBC (*P* < 0.001, *P* < 0.01) and Hgb (*P* < 0.01, *P* < 0.05) compared to the control and COL groups. In addition, 5-FU diminished PLT counts compared to the control group (*P* < 0.05) (Figure 3).

Despite the increase in WBC in the COL+5-FU group compared to the 5-FU group, no statistically considerable differences were observed (Figure 3(a)). And the results showed that in the COL+5-FU group, a significant rise in the number of RBC was found in comparison to the 5-FU group (*P* < 0.05) (Figure 3(b)).

Regarding the Hgb level, the results showed a significant diminution in the COL+5-FU group compared to the control group (*P* < 0.05). However, this group did not show a statistically significant difference from the 5-FU group (Figure 3(c)).

On the other hand, despite an increase in PLT counts in the COL+5-FU group compared to the 5-FU-receiving group, this group had no statistically significant difference from the 5-FU group (Figure 3(d)).

**Figure 4:**
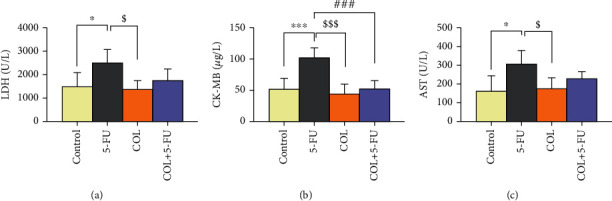
Data quantification indicates the effect of treated groups on the level of cardiac enzymes and the comparison of different groups with each other. ^∗^*P* < 0.05 and ^∗∗∗^*P* < 0.001: significant compared to the control group. ^$^*P* < 0.05 and ^$$$^*P* < 0.001: significant compared to the COL group. ^###^*P* < 0.001: significant compared to the 5-FU group. All results are expressed as the mean ± SD. LDH = lactate dehydrogenase; CK = creatine kinase; AST = aspartate transaminase; SD = standard deviation (number of animals in each group: 5).

In the following, it can be said that 5-FU administration significantly increased the levels of cardiac enzymes including LDH (*P* < 0.05, *P* < 0.05), CK-MB (*P* < 0.001, *P* < 0.001), and AST (*P* < 0.05, *P* < 0.05) compared to the control and COL groups (Figure 4).

According to the results of this study, compared to the 5-FU group, we noticed a significant decrease in the serum levels of CK-MB in the COL+5-FU group (*P* < 0.001) (Figure 4(b)).

Regarding the serum level of LDH and AST enzyme, despite the decrease in the enzyme level in the COL+5-FU group compared to the 5-FU group, no significant difference was found (Figures 4(a) and 4(c)).

### 3.4. Biochemical Analysis

**Figure 5:**
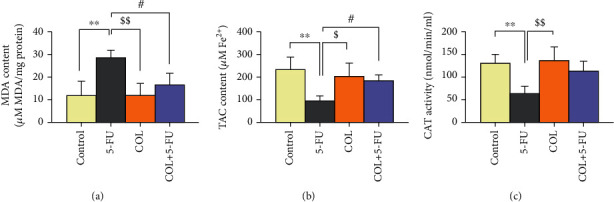
The effect of COL administration on biochemical analysis and comparison of different groups with each other. ^∗∗^*P* < 0.01: significant compared to the control group. ^$^*P* < 0.05 and ^$$^*P* < 0.01: significant compared to the COL group. ^#^*P* < 0.05: significant compared to the 5-FU group. All results are expressed as the mean ± SD. MDA = malondialdehyde; TAC = total antioxidant capacity; CAT = catalase; SD = standard deviation (number of animals in each group: 5).

The MDA levels were significantly elevated in the rats injected with 5-FU compared to the control (*P* < 0.01) and COL (*P* < 0.01) groups. However, the level of MDA in the COL+5-FU group decreased significantly compared to the 5-FU group (*P* < 0.05) (Figure 5(a)).

Administration of 5-FU significantly diminished TAC values in comparison to the control (*P* < 0.01) and COL (*P* < 0.05) groups. However, the level of TAC in the COL+5-FU group decreased significantly compared to the 5-FU group (*P* < 0.05) (Figure 5(b)).

About the CAT level, a marked decrease was observed in rats receiving 5-FU compared to the control (*P* < 0.01) and COL (*P* < 0.01) groups. Regarding the COL+5-FU group, despite an increase in CAT levels in the COL+5-FU group compared to 5-FU, this difference was not statistically substantial (Figure 5(c)).

### 3.5. Effects of COL on Histopathological Changes of Heart Tissue

**Figure 6:**
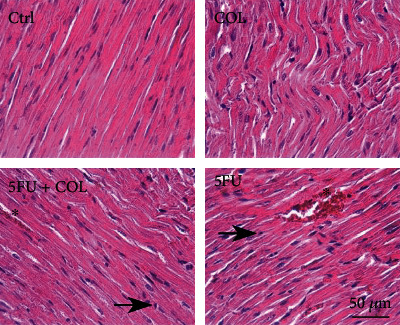
H&E staining, histopathological changes of heart tissue. Control and COL groups: normal tissue conditions. 5-FU and COL+5-FU groups: hyperemia (star) and necrosis (right arrow), magnification: ×40 (number of animals in each group: 5).

To evaluate the effect of COL on histopathological alterations of the heart, H&E staining was performed in the heart tissue. The results of H&E staining indicated that the morphology of heart cells in the control and COL groups is normal and does not show any necrosis or hyperemia. On the other hand, the 5-FU group, which received no protection, showed high levels of cardiac intoxication and prominent histopathological abnormalities, including necrosis (*P* < 0.001, *P* < 0.001) and hyperemia (*P* < 0.001, *P* < 0.001), compared with the control and COL groups (Table 4). On the other hand, levels of necrosis (*P* < 0.05, *P* < 0.05) and hyperemia (*P* < 0.01, *P* < 0.01) were observed in the COL+5-FU-receiving group in comparison to the control and COL groups. However, the group treated with COL+5-FU showed less tissue damage in terms of hyperemia compared to the 5-FU group (*P* < 0.01) (Tables 4 and 5), indicating an improvement in heart tissue damage and abnormalities by COL (Figure 6).

### 3.6. The Effect of COL on Gene Expression of COX-2 and TNF-*α*

**Figure 7:**
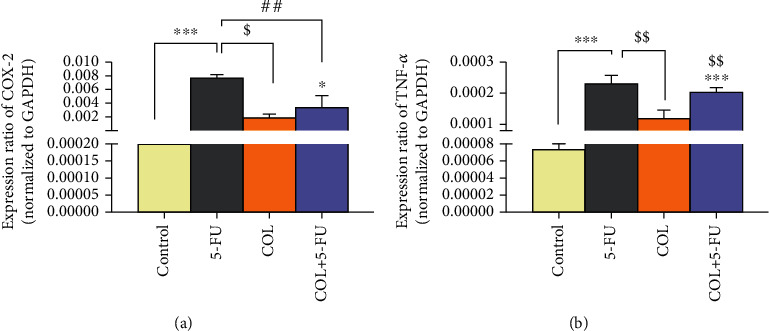
The effect of COL administration on the gene expression of COX-2 and TNF-*α* and then comparison of different groups with each other. ^∗^*P* < 0.05 and^∗∗∗^*P* < 0.001: significant compared to the control group. ^$$^*P* < 0.01: significant compared to the COL group. ^##^*P* < 0.01: significant compared to the 5-FU group. All results are expressed as the mean ± SD. COX = cyclooxygenase; TNF-*α* = *tumor necrosis factor-alpha*; SD = standard deviation (number of animals in each group: 5). COX-2 gene expression was also raised compared to the control group in the COL+5-FU (*P* < 0.05) group. However, a significant decrease in COX-2 expression was found in the COL+5-FU group (*P* < 0.01) compared to the 5-FU group (Figure 7(a)).

The results showed that there was a significant increase in the expression of COX-2 (*P* < 0.001, *P* < 0.01) and TNF-*α* genes (*P* < 0.001, *P* < 0.01) in the 5-FU group compared to the control and COL groups (Figure 7).

The results showed that in the COL+5-FU, TNF-*α* gene expression was also increased compared to the control and COL groups (*P* < 0.001, *P* < 0.01). However, no significant differences were observed between the COL+5-FU group and the 5-FU group (Figure 7(b)).

## 4. Discussion

Generally, the present study results showed that COL can raise RBC levels and body weight, also reduce the levels of CK enzyme, expression of COX-2 gene, and oxidative stress, and in addition can decrease QRS duration and ST-elevation on ECG. On the other hand, it increases the level of TAC and thus protects the heart tissue and reduces damage and abnormalities of the heart, including necrosis and hyperemia. At present, one of the basic methods for treating cancer is chemotherapy [28]. However, one of the main problems with this method is its associated side effects, including intestinal mucositis and damage to the spleen and liver, as well as the heart [29]. Many studies have been performed to reduce the side effects of these drugs. Herbal medicines can be considered to protect against the toxic effects of chemotherapy drugs [30, 31].

COL a microtubule-disrupting agent has an anti-inflammatory feature and is currently used in the treatment of inflammation-linked diseases including acute gout, Behcet's disease, and secondary amyloidosis [32, 33]. Also, Tardif et al. assessed the effectiveness of COL in preventing major adverse cardiovascular events (MACE) in patients who experienced a recent MI. They showed that using 0.5 mg of daily COL significantly reduced the risk of cardiovascular death, MI, stroke, resuscitated cardiac arrest, or urgent hospitalization for unstable angina requiring revascularization during follow-up [34].

In the present study, 5-FU-induced myocardial toxicity was evidenced by abnormal ECG changes such as enhancement of ST-elevation noted, which is itself a sign of heart injury. On the other hand, 5-FU increased the duration of ventricular depolarization (QRS duration). In line with the present study, many studies have been done. A study showed that the use of 5-FU (50 mg/kg) causes sinus tachycardia and ST-elevation and finally cardiotoxicity. Both doses of Quercetin (Q) and Rutin prevented these changes, and our findings were seen to be consistent with the literature, where treatment with flavonoids such as Q and Rutin could prevent these changes [35]. Also, the 5-FU (50 mg/kg, IP, once weekly for six successive weeks) can reduce HR and cause the prolongation in the RR interval duration as well as increase elevation in the ST [36], which is consistent with our data. In contrast, a study measuring the effect of COL on ECG parameters showed that COL (0.5 mg/kg/day) treatment had no obvious adverse effects on the sinoatrial node and atrioventricular node of rats. Therefore, COL may safely prevent atrial fibrillation (AF) vulnerability in rats [37].

In the present study, rats treated with 5-FU showed a significant reduction in body weight compared to the control group. According to previous studies, rats treated with 5-FU showed a reduction in food intake and a significant reduction in body weight which could be due to the damage to internal organs such as the liver [38] or intestine and a decrease in anaerobic bacteria in the gut [39]. However, body weight statistically increased in COL+5-FU groups when compared to the 5-FU-receiving group, which shows the beneficial role of COL in preventing weight loss due to 5-FU. One study found that the trend in weight change in COL-treated rats was dose-dependent. In fact, the administration of COL in lower doses causes weight gain. But when COL was given in higher doses to growing rats, it stopped their weight gain, and its therapeutic effect becomes a toxic effect. However, no signs of severe general toxicity, such as genitourinary and gastrointestinal bleeding, were observed [40]. In the present study, we saw a cessation of weight gain in rats treated with 5-FU, and even weight loss was observed in this group. However, treatment with COL was able to prevent severe weight loss by 5-FU to some extent.

Previous studies have shown that 5-FU toxicity is associated with oxidative damage. Actually, 5-FU induces apoptosis in rat cardiocytes through intracellular oxidative stress [41]. Since therapeutic strategies are aimed at limiting free radical-mediated cardiac injury by 5-FU, we hypothesized that COL treatment would alter cardiotoxicity induced by 5-FU. The results clearly indicate that this is true as COL treatment protected against 5-FU toxicity, as assessed by ECG changes, blood factors and cardiac enzymes, oxidative damage indices, and gene expression.

Continuous administration of chemotherapy drugs such as 5-FU can cause side effects such as anemia [42]. Because RBCs have a high content of polyunsaturated fatty acids and high levels of Hgb, they can easily be exposed to oxidative damage and can be used as a model for examining oxidative damage in biological membranes. On the other hand, erythrocyte lipid peroxidation can be associated with cell aging. Therefore, it makes sense to suggest the use of antioxidants such as COL in foods to protect blood cells from oxidative damage caused by free radical-related diseases or the use of certain drugs [43, 44]. Other study findings in this field showed moderate thrombocytopenia at 7 days after IP administration of 5-FU (150 mg/kg) and stable reversible thrombocytosis from 11 to 17 days after 5-FU injection. In fact, increased PLT production after 5-FU is associated with concomitant stimulation of the megakaryocyte-producing chamber in the rat spleen [45]. The 5-FU can also cause leukopenia, which is relieved by a glutamine-containing diet [46]. In return, COL has been shown to limit neutrophil-induced inflammation and reduce the level of inflammatory mediators in several cases [47].

Following the findings mentioned above, we found that WBC, RBC, and PLT counts, as well as Hgb levels in the 5-FU group, were significantly reduced. However, the COL-treated group significantly prevented the reduction of blood factors induced by the 5-FU.

The present results showed that administration of 5-FU increased LDH, CK-MB, and AST enzyme levels. Because these enzymes are so abundant in the heart, they can be used as a momentous indicator to identify heart damage. We also found that COL protects heart tissue against 5-FU-induced cardiac toxicity represented by a significant reduction in CK-MB serum levels. In line with the present study, a study showed that 5-FU injection increased AST, alanine aminotransferase (ALT), and CK in 5-FU-induced cardiotoxicity in rats. [48]. It can be said that an increase in serum cardiac enzymes can be due to myocardial cell damage which ultimately increases the leakage of these serum enzymes. In contrast, COL (1 mg/kg, intraperitoneally) reduces circulating levels of CK and LDH in rats with IR, and as a result, it reduces IR-induced skeletal muscle injury in rats [49].

The main cause of 5-FU-induced cardiotoxicity is not fully understood. One of the possible hypotheses for cardiac toxicity of 5-FU is oxidative stress [50]. Oxidative stress is actually a disturbance in the balance between removal and production of reactive oxygen species (ROS). ROS can cause modifications and irreversible damage to proteins, nucleic acids, and macromolecules [51] leading to stimulation of inflammatory mechanisms and cell damage [52]. 5-FU-induced cardiotoxicity can be associated with free radical damage to the myocardium [1, 53]. Drugs such as 5-FU can produce large amounts of free radicals, and the accumulation of these substances can lead to cytotoxicity and eventually lipid peroxidation in membranes and cell death [38]. Since the end product of cell membrane lipid peroxidation is MDA, its concentration indicates the severity of lipid peroxidation [54]. TAC, on the other hand, represents resistance to cell oxidative processes. Sengul et al. showed that 5-FU increased MDA levels and reduced the activities of superoxide dismutase (SOD) and glutathione (GSH) [22]. Therefore, in this study, we evaluated MDA, TAC, and CAT levels to determine the role of oxidative stress in cardiotoxicity induced by 5-FU. The present study showed that 5-FU administration increases MDA levels and also decreases TAC and CAT levels. On the other hand, the administration of COL reduced MDA levels and on the other hand increased the level of TAC. These results are consistent with previous studies [55], and the use of antioxidants such as COL minimizes ROS and cell damage. As one study showed, COL (5 days of oral 1 mg/kg) decreased the MDA level and increased CAT activity and also reduced ovarian ischemia-reperfusion (IR) injury in the experimental rat ovarian torsion model, which these findings indicate the antioxidant and anti-inflammatory activity of COL [44]. In addition, COL had favorable effects on inflammation and oxidative stress markers in an animal model of bronchopulmonary dysplasia (BPD) (hyperoxia groups were exposed to >95% oxygen for 10 days). It increased the SOD and glutathione peroxidase (GSH-Px) activities and also decreased the MDA level [56]. Also, the addition of COL (1.5 gr) to paracetamol (APAP) (daily for six months) showed that this combination lowers whole blood MDA which is a lipid peroxidation compound and elevates TAC levels in patients with knee osteoarthritis (OA). This may show the probable disease-modifying effect of COL on knee OA [57]. In addition, pretreatment with COL (300 *μ*g/kg for 7 days) reduced lipid peroxidation levels and serum *γ*-glutamyl transpeptidase (*γ*-GTP) activity. It can be concluded that COL protects the liver against acetaminophen (APAP) poisoning, possibly through its antioxidant properties, possibly acting as a free radical scavenger [58].

In addition, evaluating the histopathological changes in cardiac tissue supports the results obtained from biochemical analyses.

A histopathological study of 5-FU-induced cardiotoxicity was performed on albino rats, and multiple interstitial myocardial hemorrhages, inflammatory reactions, multifocal myofibre necrosis, vascular changes, pericarditis, and valvulitis were observed especially in the left ventricle [59]. The results of the histopathological analysis of our study indicated degenerative changes including necrosis and hyperemia in rat cardiac tissue followed by 5-FU treatment. In accordance with the findings of the ECG of rats, necrosis may be a sign of stroke in rats receiving 5-FU. Treatment with COL reduced cardiac tissue damage including necrosis and hyperemia which could be attributed to the protective and anti-inflammatory effects of COL.

Our results are consistent with the previous finding which evaluated the effect of COL on ovarian ischemia and show that IR injury decreases in rats pretreated with COL in the experimental rat ovarian torsion model [44]. In addition, Ozdemir et al. showed that COL treatment had antioxidant and anti-inflammatory effects on decreasing hyperoxic lung injury produced in neonatal rats [56]. There is evidence that several molecular mechanisms may contribute to the protective function of the heart. However, the exact mechanism of action of COL on 5-FU toxicity is not yet known.

Recent studies have shown that COL has anti-inflammatory properties and can downregulate the production of some inflammatory factors such as TNF-*α*, IL-1*β*, and IL-6 [21, 49, 56]. Also, treatment with COL (0.5 mg kg^−1^·day^−1^, via oral gavage for 3 days) inhibited postoperative atrial fibrillation (POAF) promotion in sterile pericarditis (SP) rats. These beneficial effects are probably linked to the inhibition of IL-1*β*-induced IL-6 release and subsequent atrial fibrosis. Actually, COL inhibits IL-1*β*-induced IL-6 release by suppressing the activation of P38, JNK, Akt, and NF*κ*B [37]. In line with the mentioned articles, in the molecular part of our study, in the COL+5-FU treatment group, the COX-2 levels decreased compared to the 5-FU group, which indicates the anti-inflammatory properties of COL. The COX-2 is a proinflammatory enzyme, and its expression is triggered by a number of stimulants such as hypoxia or free radical presence [60]. The COX-2 role in cardiac injury is controversial [61]. 5-FU resulted in increased expression of COX-2 in heart tissue leading to increased production of ROS [62]. Doxorubicin- (DOX-) induced COX-2 expression in cardiac tissue has been reported in different studies which could demonstrate the COX-2 role in DOX-induced cardiac damage [61]. Similarly, the findings of Ibrahim et al. and Delgado et al. showed that COX-2 inhibition ameliorated cardiotoxicity and heart failure induced by DOX, respectively [61, 63]. On the other hand, some studies reported that increased COX-2 expression levels in cardiac tissue induced by DOX protected cardiac cells against apoptosis, and COX-2 inhibition was associated with exacerbation of cardiac injury [61]. Dowd et al. announced that inhibition of COX-2 deteriorated cardiac injury induced by DOX [64]. There are several reports about the anti-inflammatory effects of COL. COL may modulate the activity of the cyclooxygenases COX-1 and COX-2, which play a major role in prostaglandin production. This hypothesis rests on the similarly prompt effect of COL and COX-2 inhibitors on gout attacks. Recent studies show that COL does not inhibit COX-1 or COX-2 in neutrophils but instead induces the COX-1 and COX-2 genes at the early stages of osteogenesis and apoptosis [65, 66]. Therefore, COL has been considered one of the most effective medications for alleviating crystal-induced joint inflammation [11, 32].

Another important proinflammatory cytokine is TNF-*α*. The 5-FU induced many types of cytokines such as TNF-*α* and b and IL-1, 6, and 12 [67]. In return, it is proven that COL modulates the TNF-*α* function. COL has been reported to reduce the release of inflammatory cytokines such as TNF-*α*, because elevated levels of proinflammatory cytokines, including TNF-*α*, are involved in IR-induced muscle damage. Therefore, COL can reduce damage to the myocardium and several other tissues. Also, these microtubules are needed to activate neutrophils in response to various stimuli, and these neutrophils play an important role in skeletal muscle damage due to IR. Neutrophils, on the other hand, are associated with the proliferation of ROS as well as proinflammatory cytokines such as TNF-*α* and IL-1*β*. It also reduces the peroxidation of inflammatory lipids and cytokines [16, 49].

Finally, according to previous studies, our results showed that COL could reduce COX-2 expression and to some extent TNF-*α* expression in the treatment group compared to the 5-FU group. Although many studies have been conducted on the protective effects of some antioxidants on the cardiotoxicity of 5-FU, the present study is the first to demonstrate the cardiac protection effects of COL against 5-FU-induced heart damage in rats.

## 5. Conclusion

In conclusion, the present study and its results showed that treatment with COL has cardiac protection effects, therapeutic. COL prevents severe weight loss due to 5-FU. In addition, COL has been shown to increase blood factor levels including RBC and transduce the level of serum enzyme of CK-MB in the heart. Also, it can reduce the expression of the COX-2 gene in the heart. COL could cause a decrease in the ST segment and QRS duration in the ECG and protect the heart through biochemical changes including decreasing MDA and increasing TAC and also histological changes such as reducing hyperemia. These data could be useful to better explore the benefits of COL as a new treatment strategy for protecting the heart against chemotherapy drugs.

## Figures and Tables

**Table 1:** Primer sequences of COX-2, TNF-*α*, and the housekeeping genes.

Primer	5′-3′
COX-2 FW	CAACCAGCAGTTCCAGTATCAGA
COX-2 RV	CAAGGAGGATGGAGTTGTTGTAGAG
TNF-*α* FW	AAATGGGCTCCCTCTCATCAGTTC
TNF-*α* RV	TCTGCTTGGTGGTTTGCTACGAC
GAPDH FW	CTACATGGCCTCCAAGGAGTAAG
GAPDH RV	CCTCCTCTTCTTCGTCTATGGC

**Table 2:** Effect of COL on 5-FU-induced changes in ECG findings.

Groups	ST-elevation (mV)	QTc duration (ms)	QRS duration (ms)	RR interval (ms)	HR (bpm)
Control	0.282 ± 0.05	116.3818 ± 7.20	12.422 ± 0.32	165.736 ± 28.33	307.195 ± 41.30
5-FU	0.46 ± 0.01^b,c^	124.01 ± 7.13	14.57 ± 0.10^b,d^	237.10 ± 16.35	263.84 ± 6.82
COL	0.326 ± 0.01	119.736 ± 29.18	12.066 ± 0.12	183.52 ± 53.67	303.812 ± 40.64
COL+5-FU	0.382 ± 0.02^a,e^	109.871 ± 23.14	11.333 ± 0.20^b,c,f^	198.953 ± 27.64	307.308 ± 42.67

^a,b^Statistically significant differences between the control and other groups (^a^*P* < 0.01, ^b^*P* < 0.001). ^c,d^Statistically significant differences between the COL and other groups (^c^*P* < 0.01, ^d^*P* < 0.001). ^k^Statistically significant differences between the 5-FU and other groups (^e^*P* < 0.05, ^f^*P* < 0.001). Values are expressed as means ± SD; *n* = 5 for each treatment group. HR = heart rate; bpm = beat per minute; SD =standard deviation.

**Table 3:** The relative weight of the heart in the treated groups compared to other groups.

Groups	Relative weight of the heart
	$\left(\frac{\text{Heart weight}}{\text{Body weight}}\right)\times 100$
Control	0.382 ± 0.01
5-FU	0.427 ± 0.01^a^
COL	0.393 ± 0.01
COL+5-FU	0.412 ± 0.03

All results are expressed as the mean ± SD. ^a^*P* < 0.01: significant compared to the control group. SD = standard deviation (number of animals in each group: 5).

**Table 4:** The effect of different treated groups on histopathological changes of heart tissue in rats and then comparisons of different groups with each other.

Groups	Histopathological changes	
	Necrosis	Hyperemia
Control	−	−
5-FU	++	+++
COL	−	−
COL+5-FU	+	+

**Table 5:** Histopathological evaluation of heart in rats treated with COL.

Parameter	Experimental groups				
	Control	5-FU	COL	COL+5-FU	*P* value^∗^
Necrosis	7.39 ± 0.92	13.6 ± 1.33^c,f^	7.88 ± 1.82	11.31 ± 2.25^a,d^	*P* ≤ 0.001
Hyperemia	7.62 ± 2.02	14.34 ± 1.48^c,f^	7.12 ± 2.29	11.12 ± 3.94^b,e,g^	*P* ≤ 0.001

Values are expressed as means ± standard deviation (SD); *n* = 5 for each treatment group. ^∗^Asymptotic significance differences of heart lesions between treated groups were observed (*P* < 0.05; Kruskal–Wallis test). ^a–c^Statistically significant differences between the control and other groups (^a^*P* < 0.05, ^b^*P* < 0.01, and ^c^*P* < 0.001; Mann–Whitney *U* test). ^d–f^Statistically significant differences between the COL group and other groups (^d^*P* < 0.05, ^e^*P* < 0.01, and ^f^*P* < 0.001; Mann–Whitney *U* test). ^g^Statistically significant differences between the 5-FU group and other groups (^g^*P* < 0.01; Mann–Whitney *U* test).

## Data Availability

Upon request, data supporting the conclusion of our study are accessible by the corresponding author.

## Supplementary Materials

Supplementary Materials There are protective effects for colchicine (COL) against 5-fluorouracil- (5-FU-) induced cardiotoxicity through a number of ways, as this agent could improve antioxidant capacity through mediating the increased level of malondialdehyde (MDA) and raising total antioxidant capacity (TAC) and catalase (CAT) as a paramount enzyme in regulating oxidative environment. It could also deter body weight from a significant decrease. Moreover, reduced blood cell count and high levels of cardiac enzymes like lactate dehydrogenase (LDH), creatine kinase myocardial band (CK-MB), and aspartate aminotransferase (AST) during 5-FU injection were moderated through COL treatment. The increase in gene expression levels of inflammatory markers like cyclooxygenase-2 (COX-2) enzyme and tumor necrosis factor-alpha (TNF-*α*) was ameliorated through COL. To evaluate heart rhythm and electrical activity (ECG), COL seems to be a deterrent for ST segment and QRS interval to prevent them from increasing. And tissue degradation like the hyperemia and necrosis triggered through 5-FU injection at rats waned after treating by COL.
