# Supplementary figures and images for: Colchicine Ameliorates 5-Fluorouracil-Induced Cardiotoxicity in Rats
Source: Oxid Med Cell Longev. 2022 Jan 28;2022:6194532. doi: 10.1155/2022/6194532 (PMC8816577; doi:10.1155/2022/6194532)

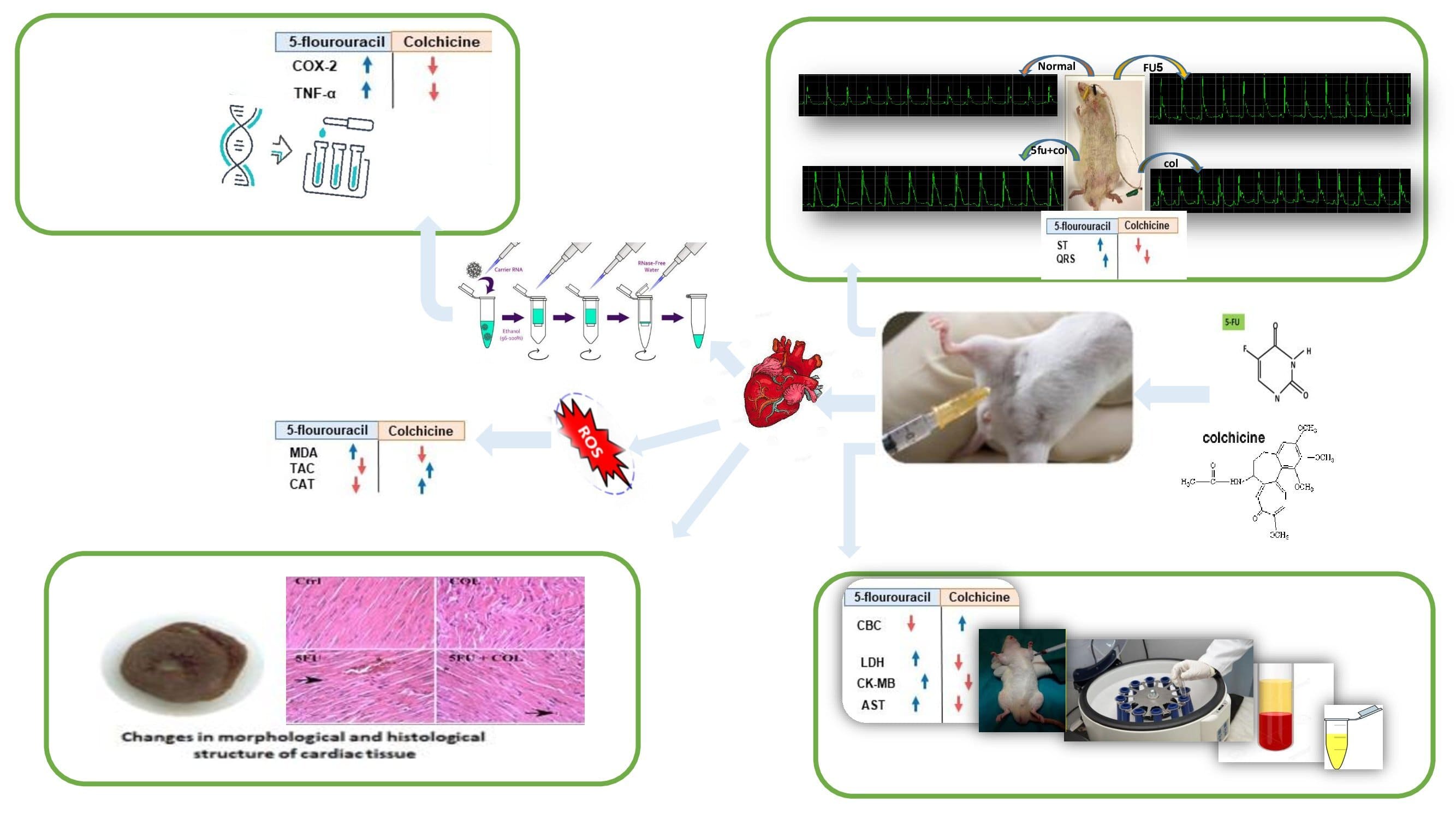

Supplement: Supplementary Materials — There are protective effects for colchicine (COL) against 5-fluorouracil- (5-FU-) induced cardiotoxicity through a number of ways, as this agent could improve antioxidant capacity through mediating the increased level of malondialdehyde (MDA) and raising total antioxidant capacity (TAC) and catalase (CAT) as a paramount enzyme in regulating oxidative environment. It could also deter body weight from a significant decrease. Moreover, reduced blood cell count and high levels of cardiac enzymes like lactate dehydrogenase (LDH), creatine kinase myocardial band (CK-MB), and aspartate aminotransferase (AST) during 5-FU injection were moderated through COL treatment. The increase in gene expression levels of inflammatory markers like cyclooxygenase-2 (COX-2) enzyme and tumor necrosis factor-alpha (TNF-α) was ameliorated through COL. To evaluate heart rhythm and electrical activity (ECG), COL seems to be a deterrent for ST segment and QRS interval to prevent them from increasing. And tissue degradation like the hyperemia and necrosis triggered through 5-FU injection at rats waned after treating by COL. [file 6194532.f1.jpg]
